# N-Lactoyl amino acids: insights from metabolite genome-wide association studies and phenome-wide association analysis

**DOI:** 10.1093/hmg/ddaf152

**Published:** 2025-09-28

**Authors:** Asma A Elashi, Aleem Razzaq, Najeha Anwardeen, Khaled Naja, Mashael Alshafai, Ilhame Diboun, Omar Albagha, Mohamed A Elrayess

**Affiliations:** Biomedical Research Center, QU Health, Qatar University, Doha P.O. Box 2713, Qatar; Biomedical Research Center, QU Health, Qatar University, Doha P.O. Box 2713, Qatar; Biomedical Research Center, QU Health, Qatar University, Doha P.O. Box 2713, Qatar; Biomedical Research Center, QU Health, Qatar University, Doha P.O. Box 2713, Qatar; Biomedical Research Center, QU Health, Qatar University, Doha P.O. Box 2713, Qatar; Department of Biomedical Sciences, College of Health Sciences, QU Health, Qatar University, Doha P.O. Box 2713, Qatar; Department of Human Genetics, Sidra Medicine, Doha, Qatar; Division of Genomics and Translational Biomedicine, College of Health and Life Sciences, Hamad Bin Khalifa University, Doha P.O. Box 34110, Qatar; Centre for Genomic and Experimental Medicine, Institute of Genetics and Cancer, University of Edinburgh, Edinburgh EH4 2XU, United Kingdom; Biomedical Research Center, QU Health, Qatar University, Doha P.O. Box 2713, Qatar; College of Medicine, QU Health, Qatar University, Doha P.O Box 2713, Qatar

**Keywords:** N-Lactoyl amino acids, mGWAS, PheWAS, Metabolomics

## Abstract

N-lactoyl-amino acids (Lac-AA) are emerging as important metabolites with diverse physiological roles. This study integrates metabolomics and genomics to investigate the genetic determinants and clinical relevance of three Lac-AA: N-Lactoyl phenylalanine (Lac-Phe), N-Lactoyl tyrosine (Lac-Tyr), and N-Lactoyl valine (Lac-Tyr). We conducted a metabolome-wide association study (mGWAS) on 2811 participants followed by a phenome-wide association study (PheWAS) and pathway enrichment analysis. Our mGWAS revealed modest genetic contributions to Lac-AA levels, with genome-wide significant loci identified for Lac-Tyr and Lac-Val, but not for Lac-Phe. PheWAS analysis linked these genetic variants to key clinical traits, including white blood cell count, platelet count, and glucose levels. Pathway enrichment highlighted the involvement of Lac-AA in immune-metabolic crosstalk, particularly in inflammation and energy metabolism. These findings suggest that Lac-AA levels are primarily influenced by dynamic metabolic or inflammatory states rather than fixed genetic factors. Our results underscore the potential of Lac-AA as metabolic sensors and biomarkers at the intersection of cellular energy states and systemic inflammation, opening new avenues for research in metabolic and inflammatory disorders.

## Introduction

N-lactoyl-amino acids (Lac-AA) is a prevalent group of metabolites found across mammalian organisms that have emerged as a major research focus in recent studies. This interest stems from both their systemic abundance and possible functional significance in biological mechanisms [1]. Their increasing recognition in recent research underscores their potential roles in metabolism and disease mechanisms. Lac-AA demonstrate a strong positive correlation with one another [2, 3], suggesting that they act as a cohesive family.

Elevated levels of Lac-AA were reported to be significantly increased in patients with mitochondrial encephalomyopathy lactic acidosis and stroke-like episodes [3], phenylketonuria, maple syrup urine disease [1], rosacea [4], and diabetic retinopathy [5]. Increased levels of Lac-Phe are associated with physical activity [6], which may suppress feeding and obesity, and influence systemic energy balance [7, 8]. Moreover, metformin treatment was shown to have a profound effect on increasing Lac-Phe levels in both mice and humans, which in turn mediates appetite-suppressing and weight-lowering properties [2, 9].

Multiple genome wide association studies have shown SNPs in CNDP2 gene associated with obesity and diabetes complications [10, 11], however, no study has been reported genetic predisposition to altered Lac-AA blood levels.

In the current study, we aim to investigate novel genetic determinants of three Lac-AA and their role in metabolic regulation through a metabolite genome-wide association study (mGWAS), and then identify their associations with various clinical traits through a phenome-wide association study (PheWAS).

## Results

### Study cohort characteristics

Our study cohort comprised 13 728 participants with comprehensive genotypic and phenotypic data. Of these, a subset of participants (*n* = 2811) had their serum samples subjected to metabolomic profiling and were used to conduct mGWAS. The clinical parameters for this study cohort (*n* = 2811) are shown in Table 1. Lac-Phe measurements were only available for 2805 participants. This study comprises approximately equal numbers of male (50.8%) and female (49.2%) participants with a mean age of 39.35 years. Majority of participants are overweight (25 to 29.9 kg/m^2^) or obese (30 kg/m^2^) with an average BMI of 29.02 kg/m^2^.

**Table 1:** Clinical characteristics of study cohort utilized for metabolite genome-wide association study. Descriptive statistics are presented as mean ± SD.

**Number of Subjects**	2811
**Gender** M/F	1428/1383
**Age**	39.35 ± 12.1
**BMI** (kg/m^2^)	29.02 ± 5.96
**Glucose** (mmol/L)	5.79 ± 2.35
**C-Peptide** (ng/ml)	2.88 ± 1.92
**HbA1c** (%)	5.75 ± 1.16
**Insulin** (U/ml)	17.14 ± 25.58

### mGWAS identifies genetic association with lac-AA

Metabolite Genome-Wide Association Study utilizing a linear mixed model was conducted separately for each of the three Lac-AA metabolites: Lac-Phe, Lac-Tyr, and Lac-Val, while accounting for age, gender, genetic principal components PC1-PC4, and metformin levels for the 2811 participants. Metformin has been associated with Lac-Phe levels, and considering that all three metabolites originate via similar mechanism [1], metformin was considered as a confounder for all analyses.

**Figure 1:**
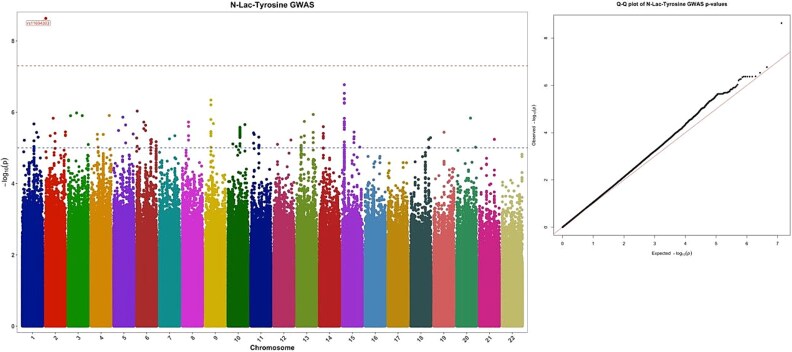
a) Manhattan plot of lac-Tyr showed genome wide significance of rs11694303 on chr2. -log_10_ p-values were plotted on Y-axis while chromosomes on X-axis. b) Quantile-quantile (QQ)-plot for mGWAS of lac-Tyr, Y-axis showed the -log10 *P*-values of observed chi square, while X-axis showed the -log10 p-values of expected chi square distribution. Black dashed line represents the genome-wide significant threshold (P-value < 5 × 10^−8^).

Lac-Tyr showed a genome-wide significant locus on chromosome 2 (Fig. 1). The genomic inflation factor λ_GC_ for the three metabolites ranged between 1.04–1.07, demonstrating no evidence of genomic inflation. Manhattan plots for Lac-Phe and Lac-Val showed no genome-wide significant association with a *P*-value > 5 × 10^−8^ (Supplementary Figs S1 and S2).

### Identification of novel association related to lac-tyrosine

**Figure 2:**
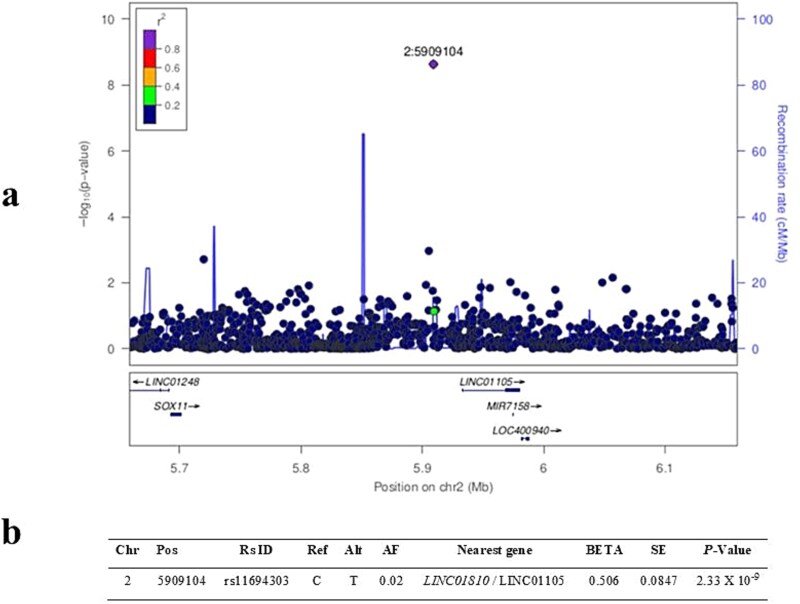
a) LocusZoom plot of the identified significant SNP (rs11694303) on chromosome 2 associated with lac-Tyr. The Y-axis (left side) represents the negative log10 p-values, and the recombination rate is shown as blue lines (right side), while the X-axis represents chromosome positions along with the nearby genes. b) Table representing the summary statistics of the novel associated SNP, including chromosome (Chr), position (Pos), alternative allele (alt), reference allele (ref), effect size (BETA), and standard error (SE).

A Novel locus, rs11694303, was identified on chromosome 2 that reached genome-wide significance (*P*-value = 2.33 × 10^−9^) for Lac-Tyr with modest effect sizes (BETA = 0.506). The associated SNP is located between long non-coding RNA genes (lncRNA), *LINC01810* (also known as *AC010729.3*) and *LINC01105*, with closer proximity to *LINC01105*. It is noteworthy that the identified locus is located outside regions previously associated with traits such as lactose metabolism and tyrosine levels. The allele frequency (AF) of rs11694303 in our study cohort was 0.02 (Fig. 2).

### Meta-analysis of lac-AA mGWAS

Meta-analyses of Lac-Phe and Lac-Val was conducted separately by integrating our GWAS summary statistics with publicly available summary statistics from a study by Chen et al. [12]. This approach led to strengthening the association of Lac-Phe and Lac-Val. Our results showed no genome-wide significant association for Lac-Phe. On the other hand, meta-analysis of Lac-Val showed genome-wide significant SNPs (*n* = 4), with the top leading SNP rs9313052 (*P*-value = 1.46 × 10^−9^) located on chromosome 5 nearest to the lncRNA encoding *LINC01019* and the protein-coding gene *IRX*. There was no evidence of heterogeneity in the identified signals, as indicated by Cochran’s Q heterogeneity statistics (Q > 0.05). Suggestive SNPs (*P*-value < 1 × 10^−6^) associated with Lac-Phe and Lac-Val are presented in Supplementary Table 1.

### Phenome-wide association analysis reveals significant associations between lac-AA and clinical traits

The genotype–phenotype associations were investigated further for 62 clinical traits in our cohort comprising of 13 728 Qatari individuals, utilizing QBB phenotypic data. Overall clinical characteristics of 62 clinical traits are presented in Supplementary Table 2.

PheWAS was conducted separately for each metabolite on SNPs associated with Lac-AA (*P*-value < 1 × 10^−6^). To ensure robustness, only those clinical traits were reported, that showed significance with Lac-AA SNPs after false discovery rate (FDR, *P*-value < 0.05) and Bonferroni corrections.

**Figure 3:**
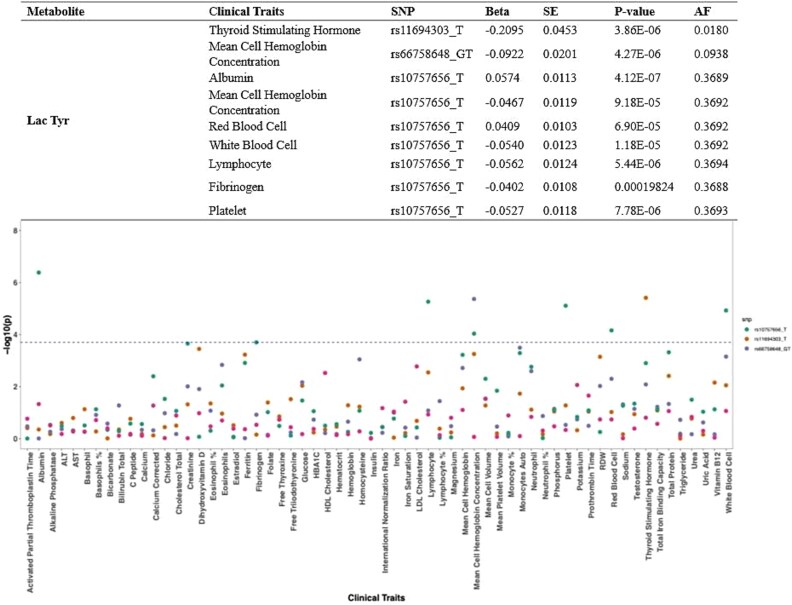
PheWAS analysis of lac-Tyr showed significant associations, including both suggestive and genome-wide significant SNPs (*P*-value < 1 × 10^−6^) with 9 clinical traits. Only three SNPs were significantly associated with multiple traits. SNP-trait association that maintained significance after FDR and Bonferroni corrections were reported. X-axis represents the clinical traits while Y-axis represents the -log_10_ of *P*-values. Effect size (BETA), and standard error (SE).

PheWAS for Lac-Tyr was performed by utilizing independent SNPs that had been obtained from our mGWAS findings (Fig. 3). The results revealed significant associations with 9 out of 62 traits, encompassing peripheral blood cells (including white and red blood cells, and platelets), fibrinogen levels, and thyroid-stimulating hormone concentrations.

**Figure 4:**
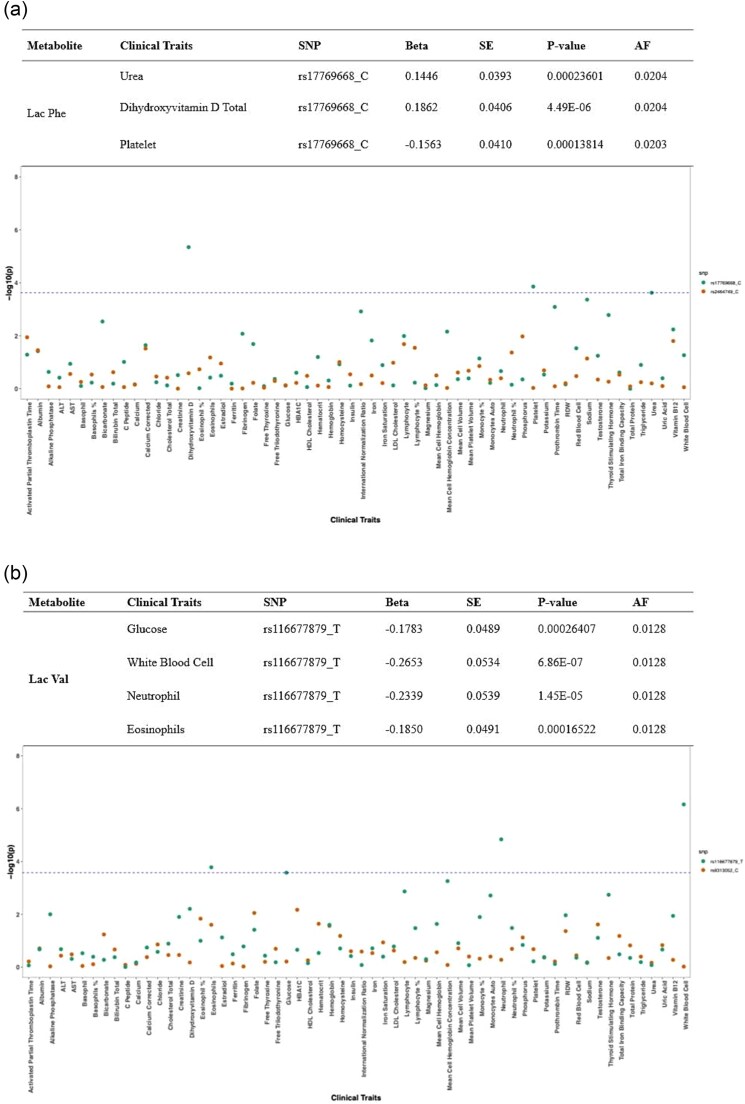
PheWAS analysis of lac-Phe (a) and lac-Val (b) showed significant association utilizing suggestively associated SNPs identified in meta-analysis (*P*-value < 1 × 10^−6^) with 62 clinical traits. Associated SNPs are presented in different colors. SNP-trait association that maintained significance after FDR and Bonferroni corrections were reported. X-axis represents the clinical traits while Y-axis represents the -log_10_ of *P*-values. Effect size (BETA), and standard error (SE).

While for Lac-Phe and Lac-Val, SNPs that were identified in meta-analysis were utilized. The analysis revealed significant associations between Lac-Phe and several clinical traits, including platelet count, Vitamin D, and urea levels, all of which exhibited large effect sizes, as shown Fig. 4a. Lac-Val demonstrated significant associations with white blood cell count, including neutrophils and eosinophils, as well as glucose levels (Fig. 4b). Overall, these results elucidated potential association of Lac-AA s to a wide spectrum of clinical phenotypes.

### Enrichment analysis identifies potential pathways associated with lac-AA

To elucidate potential biological mechanisms linked with Lac-Tyr, a less stringent *P*-value threshold (*P*-value < 1 × 10^−5^) was applied and integrated the suggestively associated SNPs of Lac-Tyr to identify relevant biological pathways.

Our analysis was conducted using overrepresentation analysis within KEGG pathways by utilizing 28 clumped independent SNPs, located within genes associated with Lac-Tyr. The results revealed two pathways potentially associated with Lac-Tyr, which reached marginal significance: the biosynthesis from tryptophan to nicotinamide adenine dinucleotide (NAD^+^) via quinolinate (adjusted *P*-value = 0.053), also known as Kynurenine pathway, and Mucin type O-glycan biosynthesis (adjusted *P*-value = 0.063). The Kynurenine pathway was significantly linked to the suggestively associated variant rs77623267 (*P*-value = 1.25 × 10^−6^), located within the *NMNAT3* gene.

Upon further investigation, we also conducted an enrichment analysis of genes by combining all independent associated SNPs (*P*-value < 1 × 10^−6^), as all Lac-AA belong to the same metabolite group. SNPs identified in the summary statistics of Lac-Tyr (*n* = 4) and SNPs identified in the meta-analysis of Lac-Phe (*n* = 2) and Lac-Val (*n* = 2) were utilized for enrichment analysis. Of the identified SNPs, only five SNPs are encoded by genes including *APBA2*, *LYRM4-AS1*, and *MOB3B* were associated with the Lac-Tyr, while *NALF1* and *BIRC3* were linked to the Lac-Val and Lac-Phe, respectively. This resulted in the identification of several significantly enriched pathways, including apoptosis, NF-kappa B signaling pathway, and TNF signaling pathway, reported in Table 2. Notably, these pathways were associated to a single gene, *BIRC3* associated with Lac-Phe (rs17769668; *P*-value = 6.65 × 10^−7^). It is imperative to note that the enrichment analysis was constrained by the small number of associated genes, which limits statistical power and warrants cautious interpretation. Gene assignment was based on physical proximity, which may not always represent the causal gene. To further investigate this, we cross-checked our lead metabolite-associated SNPs using GTEx v8; however, none showed significant cis-eQTL associations with nearby genes. Consequently, the enrichment findings should be regarded as exploratory and hypothesis-generating, requiring validation in larger cohorts and functional studies.

**Table 2:** Pathway enrichment analysis results for rs17769668, a SNP suggestively associated with lac-Phe. Multiple signaling and immune-related pathways remained significant after adjustment for multiple testing.

**Pathway**	**P-value**	**Adjusted P-value**
NF-kappa B signaling pathway	0.012	0.025
TNF signaling pathway	0.014	0.025
Apoptosis	0.016	0.025
Ubiquitin mediated proteolysis	0.017	0.025
Hippo signaling pathway	0.018	0.025
NOD-like receptor signaling pathway	0.022	0.025

## Discussion

Integrating metabolomics and genomics enhances our understanding of complex biological pathways, improves identification of causal genes, and provides deeper insights into the functional relationships between genetic variants, metabolites, and disease phenotypes. N-lactoyl-amino acids have emerged as a significant class of metabolites, playing diverse physiological roles that span across various biological processes and body systems [1, 2, 7, 9].

In this study, we first conducted a metabolite genome-wide association study to identify SNPs significantly associated with three Lac-AA, thereby elucidating their genetic determinants. These SNPs are then leveraged in a phenome-wide association study to systematically explore their associations with diverse clinical phenotypes. An enrichment analysis was also performed to identify overrepresented metabolic pathways linked to the genetic regulation of Lac-AA.

Our results revealed modest genetic contributions to Lac-AA metabolites. For Lac-Tyr, the novel genome-wide significant locus (rs11694303) exhibited a moderate effect size (BETA = 0.506) and low allele frequency in our cohort (AF = 0.02). Similarly, the meta-analysis for Lac-Val showed genome-wide signals, but with effect sizes comparable to Lac-Tyr. Lac-Phe revealed no genome-wide associations, even with the meta-analysis. The lack of strong, replicated loci across all the three studied metabolites, combined with the absence of heterogeneity in meta-analysis, may suggest that non-genetic factors such as environmental exposures, pathological conditions, and pharmacological interventions play a more important role in influencing the levels of these metabolites. This is not surprising, since Lac-AA, particularly Lac-Phe, are known to increase after exercise [7], upon metformin treatment [2, 9], and in many diseases and conditions [1, 3, 13, 14]. It is important to note that the mGWAS might be underpowered to detect smaller effect sizes, and a larger sample size is required in future studies.

PheWAS analysis connects genetic associations from mGWAS to clinical relevance by linking Lac-AA metabolites to specific phenotypic traits. Our results revealed that Lac-AA are linked to important clinical traits, even if genetic predisposition does not strongly influence their levels.

Both Lac-Tyr and Lac-Val showed significant associations with WBC count. This aligns with the potential role of Lac-AA in immune regulation, as they are produced in immune cells like macrophages and monocytes [7, 9] during inflammation [4, 14]. Moreover, both Lac-Tyr and Lac-Phe exhibited significant associations with platelets count. This suggests a potential role in coagulation or vascular health, consistent with findings that link these metabolites to cardiovascular risk [6, 14]. The link of Lac-Tyr to fibrinogen, a marker of inflammation and coagulation, reinforces this possible connection to inflammation and vascular health.

The association with TSH highlights a novel intersection between Lac-Tyr and thyroid function, which warrants further exploration given the central role of thyroid hormones in metabolic homeostasis. The inverse association of Lac-Val with glucose is particularly intriguing, suggesting a potential protective mechanism against hyperglycemia or a compensatory response in metabolic dysregulation, which could have implications for diabetes research.

It is imperative to mention that these SNPs may influence biological pathways common to both metabolite regulation and observed clinical traits (pleiotropy effect), or these genetic variants might indirectly affect Lac-AA via these traits (mediation effect). Future functional studies are warranted to disentangle causality and translational relevance.

The enrichment pathway analysis further corroborates our results, positioning these metabolites as key players in immune-metabolic crosstalk. The convergence of kynurenine [15, 16], Mucin-Type O-Glycan [17], NF-κB [18], and TNF [19] pathways with PheWAS traits highlights a unifying theme: Lac-AA metabolites may serve as metabolic sensors linking cellular energy states to systemic inflammation and homeostasis. In line with these findings, a recent study by our group demonstrates that Lac-Phe disrupts insulin signaling, induces inflammation, and impairs mitochondrial respiration in cell models, further supporting the potential roles of Lac-AA metabolites in metabolic and inflammatory regulation [20]. These insights pave the way for targeted investigations into Lac-AA metabolites as biomarkers or modulators of inflammatory and hematological disorders.

Our study has several limitations which should be acknowledged. Primarily, our association study was constrained by a small sample size, which may have resulted in insufficient statistical power to detect genetic determinants with larger effect sizes. Due to this limited sample size, combined with their small effect size, we were also unable to validate our identified genome-wide association significant SNPs associated with Lac-AA in an independent cohort. Additionally, we were unable to detect significances in causation analysis of Lac-AA and their associated phenotypes through Mendelian randomization. Validation in larger cohorts is warranted to confirm the findings and establish the robustness of the associations. Further studies with increased statistical power and diverse populations are necessary to elucidate the genetic architecture underlying Lac-AA and its potential causal relationships with related phenotypes. This will help to clarify the biological mechanisms and inform potential therapeutic targets or interventions.

The levels of Lac-AA metabolites may primarily reflect dynamic metabolic or inflammatory states rather than static genetic determinants. Our findings highlight that Lac-AA metabolites are significantly associated with clinical trait and pathways, suggesting their potential role as metabolic sensors at the intersection of immune and metabolic pathways, linking cellular energy states to systemic inflammation and homeostasis. Nonetheless, the complexity of these interactions and the limitations of our study emphasize the need for further research. Future studies with larger cohorts and functional experiments are essential to validate these associations, clarify causality, and explore the translational potential of Lac-AA in clinical settings.

## Materials and methods

### Study cohort

Study participants were recruited from Qatar Biobank (QBB), a population-based cohort of native Qataris and long-term residents of ≥15 years in Qatar [21]. Prior to data and sample collection, all participants were consented. As part of recruitment, participants were asked to complete a standardized questionnaire, self-reporting information including history of diseases, medication intake, lifestyle and diet [22]. In addition, clinical measurements and biological samples including blood were obtained from all participants. This study was approved by the Institutional Review Boards of QBB (E-2024-QF-QBB-RES-ACC-00199-0267) and Qatar University (QU-IRB 215/2024-EM).

### Whole genome sequencing of QBB participants

Whole genome sequencing (WGS) was performed in 14 669 QBB participants by Qatar Precision Health Institute (QPHI) as previously described [23]. Briefly, Genomic libraries were prepared after extracting DNA from peripheral blood using Qiagen MIDI kit (Qiagen, Germany). The libraries were sequenced using illumina (USA) HiSeq X Ten with a minimum coverage of 30X at Sidra Medicine. FastQC was used to check the quality of the generated fastq files (https://www.bioinformatics.babraham.ac.uk/projects/fastqc/). The fastq files were aligned to human reference genome GRCh38 using bwa.kit (v0.7.12). Mapping quality was assessed using Picard (v1.117). Variant calling was performed using GATK 3.4 best practices to generate gVCF files. This gVCF file was checked for quality control and extensive measures were taken using PLINK (version-2.0) to get the best quality data for further analysis.

### Quality control

To ensure the quality of data, variants with genotype call rate < 90% and samples with call rate < 95% (*n* = 3) were removed. Additionally, variants with minor allele frequency (MAF) < 1%, Hardy–Weinberg *P* < 1 × 10^−6^ and variants on X chromosome were further removed. Next, samples with ambiguous gender between genetically determined and reported sex in the available data were removed (*n* = 441). Samples showing excess heterozygosity (*n* = 48) were removed. The heterozygosity rate was calculated for each sample, and the mean heterozygosity and standard deviation (SD) were then computed across all samples. Samples with heterozygosity rates more than ±4 SD from the mean were excluded. Moreover, Identity-by-decent (IBD) analysis was performed to remove related participants using independent pruned SNPs. To get independent pruned SNPs, Linkage Disequilibrium (LD) was performed on a 200 kb window with r^2^ = 0.05. Individual pairs with pi-hat value > 0.35 were considered as duplicated and removed. Furthermore, Multidimensional scaling (MDS) was performed to identify population outliers (*n* = 449). Finally, a total of 13 728 participants and 6 864 395 variants were used for further downstream analysis.

### Metabolomics

Serum samples were collected from QBB participants (*n* = 3000) and subjected to untargeted metabolomics analysis using the Metabolon platform at the Anti-Doping Lab, Qatar, following a previously described protocol [24]. Metabolic profiling was performed using a Waters ACQUITY ultra-performance liquid chromatography (UPLC) system (Waters Corporation, Milford, MA, USA) coupled with a Thermo Scientific Q-Exactive high-resolution/accurate mass spectrometer (Thermo Fisher Scientific, Inc., Waltham, MA, USA). The mass spectrometer was equipped with a heated electrospray ionization (HESI-II) source and an Orbitrap mass analyzer operated at 35000 mass resolution. In brief, protein components were extracted from serum samples using methanol and divided into five fractions for comprehensive metabolite profiling using multiple chromatographic and ionization methods, as previously reported [24]. Raw data underwent extraction, peak identification, and quality control processes using Metabolon’s proprietary hardware and software [25]. The resulting metabolomic data were cross-referenced against library entries containing over 3300 pure standard compounds. Library matches for each compound were carefully examined and corrected when necessary to ensure accurate metabolite identification.

### Metabolite genome-wide association studies (mGWAS)

Scalable and Accurate Implementation of Generalized mixed model (SAIGE), an R package, was used to perform generalized linear mixed models [26]. SAIGE effectively and efficiently performs association tests on a large sample size while considering sample relatedness using the saddle point approximation [26]. The variance ratio in SAIGE is the ratio of the variance of score statistics calculated for a subset of randomly selected genetic variants, estimated both with and without incorporating random effects such as sample relatedness in the model. SAIGE utilizes this ratio to adjust the variance of score statistics in the association tests for all variants, thereby accounting for relatedness and population structure while maintaining computational efficiency.

A set of 51 967 LD pruned SNPs were used to estimate variance ratio (VR). The VR was then used in the second step to perform association tests. In mGWAS, the model was adjusted with covariates including age, gender, and principal components (PC1-PC4). The PCs were calculated based on the genotype data. PLINK was used to perform principal component analysis, and the first four principal components were included as covariates based on the eigenvalues, as described previously [23]. Since Lac-Phe levels were affected by metformin intake, the model was also adjusted for metformin uptake users as a covariate [2, 9]. Genome-wide significant association threshold (*P* < 5 × 10^−8^) was applied to identify statistically significant associations. The mGWAS for Lac-Phe (*n* = 2805), Lac-Val (*n* = 2811), and Lac-Tyr (*n* = 2811) in the Qatari population was analyzed, respectively. The study’s focus on only three Lac-AA metabolites was dictated by the availability of QBB metabolomics data, which was restricted to these specific metabolites.

### Genome-wide association meta-analysis

A meta-analysis was conducted utilizing summary statistics from our mGWAS findings on Lac-Phe and Lac-Val, combined with summary statistics from a recently published dataset of 1091 plasma metabolites by Chen et al. [12]. Our analysis focused on two Lac-AA metabolites of interest, as the association for Lac-Tyr was not included in Chen et al. study. This study tested the association across four distinct population groups, comprising 8299 European, 108 South Asian, 104 East Asian, and 60 African individuals. Summary statistics for these cohorts were obtained from the NHGRI-EBI Catalog of human genome-wide association studies. The meta-analysis was performed using PLINK v1.9, employing a fixed-effects model.

### Phenome-wide association study (PheWAS)

To check the association of Lac-AA with clinical traits, PheWAS was performed using an R package PheWAS [27], which works on linear regression for continuous traits and logistic regression for binary traits while adjusting for multiple covariates. PheWAS was performed for all the three Lac-AA metabolites (continuous values) separately on 62 clinical traits of 13 728 individuals obtained from QBB. In case of Lac-Phe and Lac-Val, suggestively associated (*P*-value < 1x10^−6^) SNPs were obtained from the meta-analysis, while Lac-Tyr SNPs (*P*-value < 1×10^−6^) were obtained from our mGWAS findings. The resulting SNPs were clumped (r^2^ = 0.01) using PLINK v1.9, and final list of SNPs (Lac-Phe; *n* = 2, Lac-Val; *n* = 2, Lac-Tyr; *n* = 4) were used to perform PheWAS. The traits involved in this study were normalized using rank-based inversed normalization method prior to perform PheWAS analysis using R. To perform PheWAS analysis, covariates including age, gender, BMI, and fasting time were adjusted.

### Pathway enrichment analysis

The genetic variants (*P*-value <1 × 10^−6^) from the current mGWAS Lac-Tyr study, along with the Lac-Phe and Lac-Val meta-analysis SNPs were utilized to perform KEGG over-representation analysis. LD clumping was performed using a threshold of r^2^ > 0.01 and excluded variants within a 1 Mb distance from the index SNPs for each metabolite to identify independent SNPs. These clumped SNPs were then mapped to genes using various annotation datasets, including the Bioconductor annotation data package for Human (org.Hs.eg.db), Ensembl, and UCSC. The resulting gene list was used as input for over-representation analysis using the enrichKEGG function in the *clusterProfiler* R package [28]. The adjusted *P*-values from this analysis (*P*-value < 0.05) was considered significant.

## Supplementary Material

Figure_S1_ddaf152

Figure_S2_ddaf152

Supplementary_Tables_ddaf152

## Data Availability

The datasets used and/or analyzed during the current study are available from the corresponding author on reasonable request.

## References

[1] Jansen RS, Addie R, Merkx R. et al. N-lactoyl-amino acids are ubiquitous metabolites that originate from CNDP2-mediated reverse proteolysis of lactate and amino acids. Proc Natl Acad Sci USA 2015;112:6601–6606. 10.1073/pnas.1424638112. 25964343 PMC4450436

[2] Scott B, Day EA, O’Brien KL. et al. Metformin and feeding increase levels of the appetite-suppressing metabolite lac-Phe in humans. Nat Metab 2024;6:651–658. 10.1038/s42255-024-01018-7 PMC11052712 38499765

[3] Sharma R, Reinstadler B, Engelstad K. et al. Circulating markers of NADH-reductive stress correlate with mitochondrial disease severity. J Clin Invest 2021;131:e136055. 10.1172/JCI136055. PMC7810486 33463549

[4] Yao H, Shen S, Gao X. et al. The causal relationship between blood metabolites and rosacea: a Mendelian randomization. Skin Res Technol 2024;30:e13796. 10.1111/srt.13796. 38895784 PMC11187845

[5] Fernandes Silva L, Hokkanen J, Vangipurapu J. et al. Metabolites as risk factors for diabetic retinopathy in patients with type 2 diabetes: a 12-year follow-up study. J Clin Endocrinol Metab 2023;109:100–106. 10.1210/clinem/dgad452. 37560996 PMC10735554

[6] Sellami M, Naja K, Almuraikhy S. et al. N-Lactoyl amino acids as metabolic biomarkers differentiating low and high exercise response. Biol Sport 2025;42:331–344. 10.5114/biolsport.2025.145912. PMC11963115 40182705

[7] Li VL, He Y, Contrepois K. et al. An exercise-inducible metabolite that suppresses feeding and obesity. Nature 2022;606:785–790. 10.1038/s41586-022-04828-5. 35705806 PMC9767481

[8] Hoene M, Zhao X, Machann J. et al. Exercise-induced N-Lactoylphenylalanine predicts adipose tissue loss during endurance training in overweight and obese humans. Metabolites 2023;13:15. 10.3390/metabo13010015. PMC9863672 36676940

[9] Xiao S, Li VL, Lyu X. et al. Lac-Phe mediates the effects of metformin on food intake and body weight. Nat Metab 2024;6:659–669. 10.1038/s42255-024-00999-9. 38499766 PMC11062621

[10] Ahluwalia TS, Lindholm E, Groop LC. Common variants in CNDP1 and CNDP2, and risk of nephropathy in type 2 diabetes. Diabetologia 2011;54:2295–2302. 10.1007/s00125-011-2178-5. 21573905

[11] Yamakawa-Kobayashi K, Otagi E, Ohhara Y. et al. The combined effects of genetic variation in the CNDP1 and CNDP2 genes and dietary carbohydrate and carotene intake on obesity risk. J Nutrigenet Nutrigenomics 2017;10:146–154. 10.1159/000485798. 29402779

[12] Chen Y, Lu T, Pettersson-Kymmer U. et al. Genomic atlas of the plasma metabolome prioritizes metabolites implicated in human diseases. Nat Genet 2023;55:44–53. 10.1038/s41588-022-01270-1. 36635386 PMC7614162

[13] van Wegberg AMJ, van der Weerd JC, Engelke UFH. et al. The clinical relevance of novel biomarkers as outcome parameter in adults with phenylketonuria. J Inherit Metab Dis 2024;47:624–635. 10.1002/jimd.12732. 38556470

[14] Rogers RS, Sharma R, Shah HB. et al. Circulating N-lactoyl-amino acids and N-formyl-methionine reflect mitochondrial dysfunction and predict mortality in septic shock. Metabolomics 2024;20:36. 10.1007/s11306-024-02089-z. 38446263 PMC10917846

[15] Mándi Y, Vécsei L. The kynurenine system and immunoregulation. J Neural Transm (Vienna) 2012;119:197–209. 10.1007/s00702-011-0681-y. 21744051

[16] Tsuji A, Ikeda Y, Yoshikawa S. et al. The tryptophan and kynurenine pathway involved in the development of immune-related diseases. Int J Mol Sci 2023;24:5742. 10.3390/ijms24065742. PMC10051340 36982811

[17] Bergstrom KS, Xia L. Mucin-type O-glycans and their roles in intestinal homeostasis. Glycobiology 2013;23:1026–1037. 10.1093/glycob/cwt045. 23752712 PMC3858029

[18] Lawrence T . The nuclear factor NF-kappaB pathway in inflammation. Cold Spring Harb Perspect Biol 2009;1:a001651. 20457564 10.1101/cshperspect.a001651 PMC2882124

[19] Popa C, Netea MG, van Riel PL. et al. The role of TNF-alpha in chronic inflammatory conditions, intermediary metabolism, and cardiovascular risk. J Lipid Res 2007;48:751–762. 10.1194/jlr.R600021-JLR200. 17202130

[20] Hedaya L, Naja K, Almuraikhy S. et al. N-Lactoyl phenylalanine disrupts insulin Signaling, induces inflammation, and impairs mitochondrial respiration in cell models. Cells 2025;14:14. 10.3390/cells14161296. PMC12384308 40862774

[21] Al Thani A, Fthenou E, Paparrodopoulos S. et al. Qatar biobank cohort study: study design and first results. Am J Epidemiol 2019;188:1420–1433. 10.1093/aje/kwz084. 30927351

[22] Suhre K, Stephan N, Zaghlool S. et al. Matching drug metabolites from non-targeted metabolomics to self-reported medication in the Qatar biobank study. Metabolites 2022;12:12. 10.3390/metabo12030249. PMC8948833 35323692

[23] Thareja G, Al-Sarraj Y, Belkadi A. et al. Whole genome sequencing in the middle eastern Qatari population identifies genetic associations with 45 clinically relevant traits. Nat Commun 2021;12:1250. 10.1038/s41467-021-21381-3. 33623009 PMC7902658

[24] Al-Khelaifi F, Diboun I, Donati F. et al. A pilot study comparing the metabolic profiles of elite-level athletes from different sporting disciplines. Sports Med Open 2018;4:2. 10.1186/s40798-017-0114-z. 29305667 PMC5756230

[25] Evans A, Bridgewater B, Liu Q. et al. High resolution mass spectrometry improves data quantity and quality as compared to unit mass resolution mass spectrometry in high-throughput profiling metabolomics. Metabolomics 2014;4:1–3.

[26] Zhou W, Nielsen JB, Fritsche LG. et al. Efficiently controlling for case-control imbalance and sample relatedness in large-scale genetic association studies. Nat Genet 2018;50:1335–1341. 10.1038/s41588-018-0184-y. 30104761 PMC6119127

[27] Carroll RJ, Bastarache L, Denny JC. R PheWAS: data analysis and plotting tools for phenome-wide association studies in the R environment. Bioinformatics 2014;30:2375–2376. 10.1093/bioinformatics/btu197. 24733291 PMC4133579

[28] Wu T, Hu E, Xu S. et al. clusterProfiler 4.0: a universal enrichment tool for interpreting omics data. Innovation (Camb) 2021;2:100141. 10.1016/j.xinn.2021.100141. 34557778 PMC8454663

