# Supplementary figures and images for: N-Lactoyl amino acids: insights from metabolite genome-wide association studies and phenome-wide association analysis
Source: Hum Mol Genet. 2025 Sep 28;34(22):1865–73. doi: 10.1093/hmg/ddaf152 (PMC12581823; doi:10.1093/hmg/ddaf152)

## Slide 1
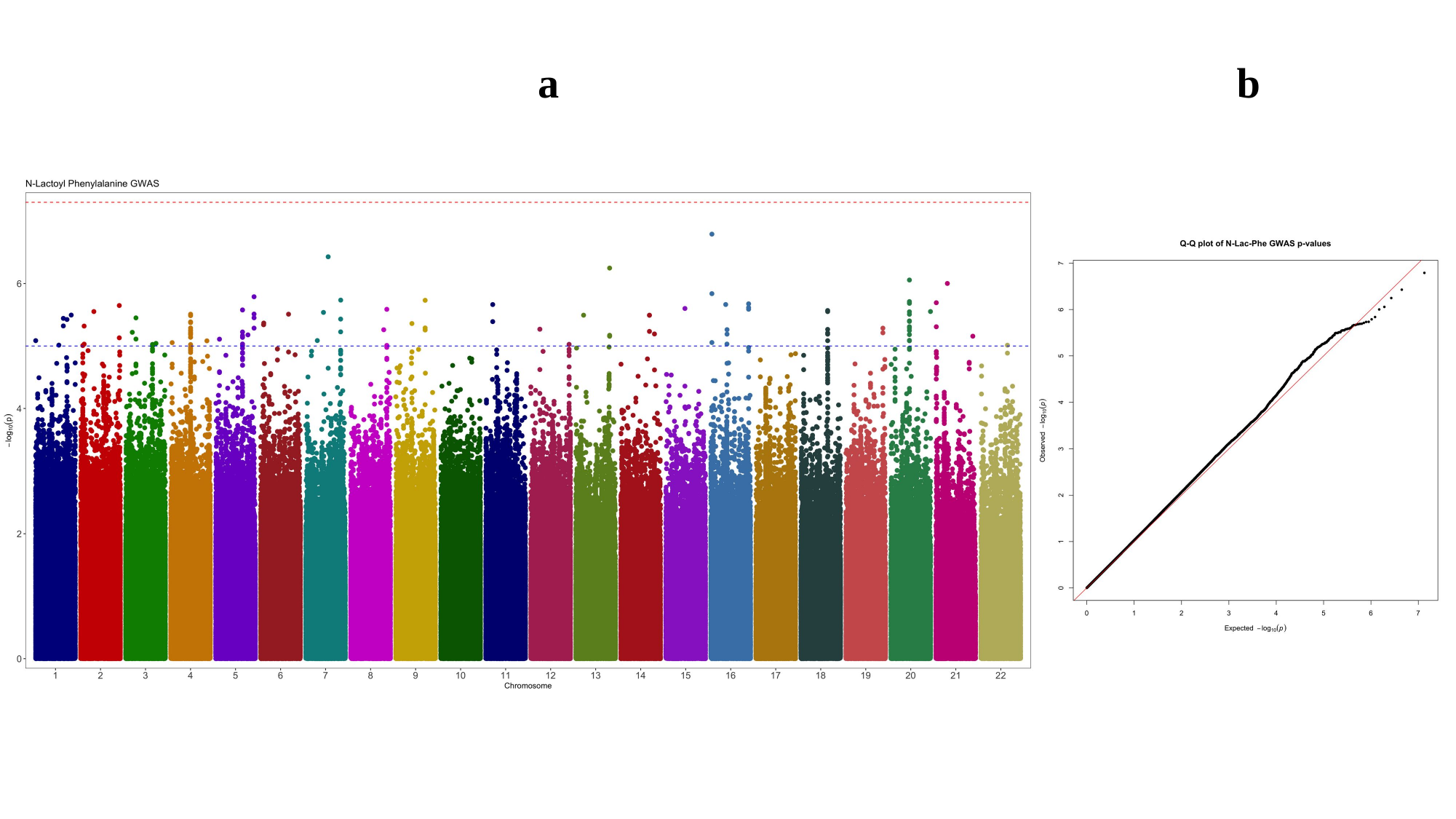

a
b

Supplement: Figure_S1_ddaf152 [file figure_s1_ddaf152.pptx]

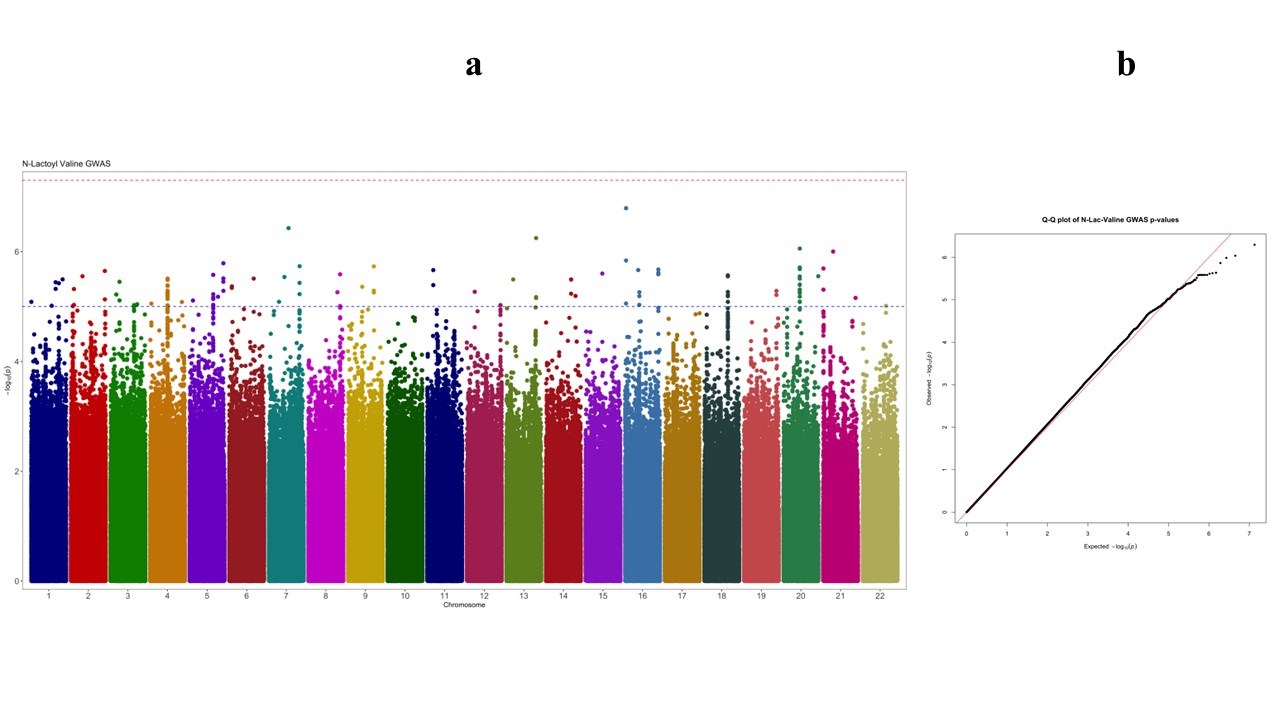

Supplement: Figure_S2_ddaf152 [file figure_s2_ddaf152.jpeg]
